# Giant aneurysm of the basilar artery in an 86 year old woman

**DOI:** 10.12688/f1000research.2-112.v1

**Published:** 2013-04-18

**Authors:** Yong peng Yu, Hong qin Zhao, Wei feng Ren, Xiang lin Chi

**Affiliations:** 1 Department of Neurology, Affiliated Wendeng Center Hospital of Weifang Medical College, Weihai, 264400, China; 2 Department of Neurology, Affiliated Hospital of Qingdao University Medical College, Qingdao, 266000, China; 3 Intensive Care Unit, Affiliated Hospital of Qingdao University Medical College, Qingdao, 266000, China

## Abstract

In this article we present an 80 year old female patient with an unruptured giant aneurysm of the basilar artery presenting with posterior circulation ischemic symptoms. Angiography and CT revealed giant basilar aneurysmal dilatation with severe and wide intracranial arteriosclerosis. We described the uniqueness of this case. Giant basilar aneurysm is associated with various complications particularly brain stem infarction. It is emphasized that arteriosclerosis plays an important role in the formation of giant basilar aneurysms.

## Introduction

Giant basilar aneurysm is a rare condition with elevated mortality within a few days of onset if untreated. On the basis of clinical course, a giant aneurysm may be categorized as a chronic type which grows relatively slowly, and may lead to serious complications such as cerebral ischemia or subarachnoid hemorrhage ^1^. We report a case of an 80-year-old woman with a surgically untreated giant basilar aneurysm. The patient presented ischemic events involving posterior circulation without aneurysmal rupture or bleeding.

## Case report

**Figure 1.**
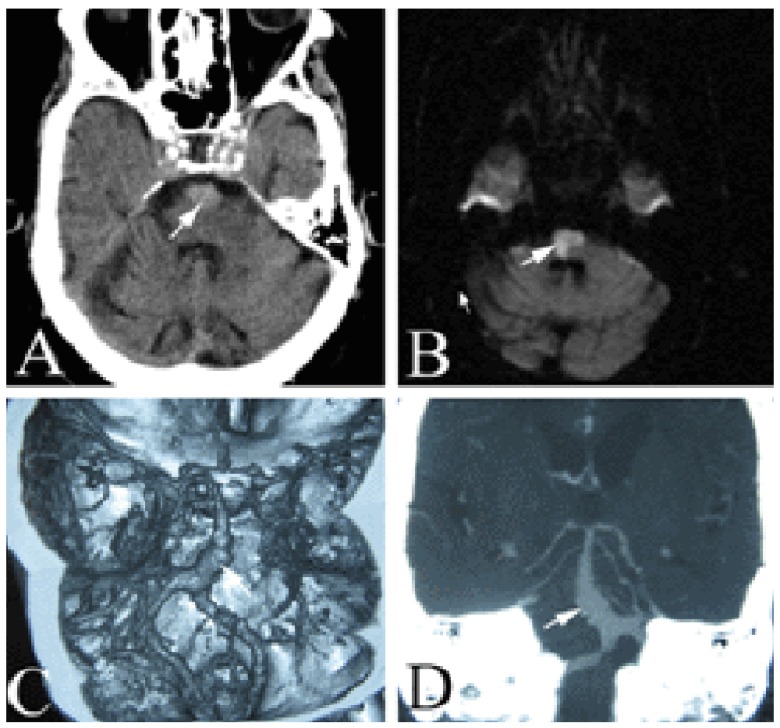
( **A**) CT scan of the brain showing an area of high density in the front of the pons (arrow) similar to a pontine hemorrhage. ( **B**) Brain MRI revealed acute brainstem infarction (arrow). ( **C**, **D**) CT angiography of the head showed a giant aneurysm of the basilar artery (27 mm by 10 mm) (arrow).

An 86-year-old woman who had a 10-year history of hypertension was admitted to the hospital with slurred speech, diplopia, vomiting and left limb paralysis for one hour. On physical examination she was afebrile, her heart rate was 72 beats per minute, her blood pressure 170/110 mmHg, and her oxygen saturation 96%. The neurological state at acceptance was: dysarthria, right conjugate gaze palsy, pseudobulbar palsy, positive bilateral Babinski sign and grade zero (Oxford Scale) in muscle strength of the left limbs. Computed tomography (CT) of the brain showed an area of high density in the front of the pons ( Figure 1A) which was very similar to a pontine hemorrhage. Magnetic resonance imaging (MRI) scans of the brain revealed acute brainstem infarction ( Figure 1B), CT angiography of the head showed a giant aneurysm of the basilar artery (27 mm × 10 mm) ( Figure 1C and D). On the basis of imaging studies, the patient received a diagnosis of brainstem infarction and giant aneurysm of the basilar artery, most likely secondary to severe atherosclerotic arterial disease. The patient’s family declined to undergo further investigation, surgical management or endovascular intervention, citing the expense, the size of the aneurysm and the risks associated with surgical intervention as reasons. The patient instead received conservative treatment with enalapril to control blood pressure and aspirin for anti-blood platelet aggregation. Her condition improved and became stable.

## Discussion

Intracranial aneurysms are vascular abnormalities that are most commonly seen in elderly patients with severe atherosclerosis. They can occur between 35–65 years, particularly in populations with a mean age over 50 ^2^. 60% of ruptured aneurysms occur in women ^3^. Basilar aneurysms can be graded according to their diameter into small, (<12 mm), large (12–25 mm), and giant (>25 mm) ^4, 5^. Small aneurysms are usually asymptomatic, while large ones may cause distal embolism or occlusion of the perforating arteries. 60% are found in anterior circulation and 40% in posterior circulation with a predilection for the vertebrobasilar arteries ^6^. However, the cause of giant aneurysms remains elusive ^7^. Several mechanisms have been proposed for the formation of a giant aneurysm, including the effect of prolonged hemodynamic stress, roles and relationships of anatomic location, hemodynamic and degenerative factors, physical exertions and emotional stress, congenital abnormality, mechanical injury resulting from poststenotic turbulence, inflammatory vasculopathy, severe reticular fiber deficiency in the muscle layer, and intimal disruption from arterial dissection ^7, 8^. Clinically, a giant aneurysm usually presents as a space-occupying lesion with a compressive effect on posterior fossa structures, or causes posterior circulation infarction due to the occlusion of penetrating vessels, or distal embolism from the thrombus in the aneurysm lumen ^1^. Alternative mechanisms including compression, vasospasm and hemodynamic mechanisms are more sensitive to orthostatic hypotension, in cases of the poor cerebral collateral circulation ^9, 10^.

This patient was unique in several ways. First, the patient presented with a giant basilar aneurysm without a history of ischemic stroke, and had extensive and severe intracranial arterial atherosclerosis. Secondly, posterior circulation symptoms and signs such as vomiting, diplopia, dysarthria and right conjugate gaze palsy were the initial clinical manifestations with high density as showed in the crainal CT ( Figure 1A). Cranial MRI displayed brainstem infarction and computer angiography revealed a giant basilar artery aneurysm, which was not the typical spindle shape, with wide caliber in the proximal centre and more slender at the extremities. Thirdly, a freshly formed thrombus was not found in the giant basilar aneurysm as is normally the case. Aside from brainstem infarction, there were no acute ischemic lesions in other brain parenchyma. Brainstem infarction may result from occlusion of the pontine perforators due to hypertensive arteriosclerosis, which may lead to perforating artery disease ^11^. A possibility is that extrinsic compression by the expanding giant aneurysm led to occlusion of the pontine perforators, although artery-to artery embolism occurs relatively often in patients with giant basilar aneurysms ^12^.

If a cranial CT scan shows high density in the brainstem, and there is suspicion of a brainstem hemorrhage, angiography should be performed quickly to check for the presence of a giant basilar artery aneurysm. Our case is rare and emblematic because of the patient’s advanced age and the progressive growth of the untreated chronic giant aneurysm presenting with ischemic events but without history of ischemic stroke, rupture or bleeding. Severe and wide intracranial arteriosclerosis may play a pivitol role in the formation of giant aneurysms of the basilar artery.

## Consent

Written informed consent for publication of clinical details and clinical images was obtained from the patient’s next of kin.
