# Supplementary material for: Giant aneurysm of the basilar artery in an 86 year old woman
Source: F1000Res. 2013 Apr 18;2:112. [Version 1] doi: 10.12688/f1000research.2-112.v1 (PMC3814923; doi:10.12688/f1000research.2-112.v1)
Supplement: Peer review history

# Peer review: Referee response for version 1

**Published:** 2013-05-03

**Authors:** Azize Esra Gürsoy

**Affiliations:** 1 Department of Neurology, Medical Faculty, Bezmialem Vakif University, Istanbul, Turkey

I have read this submission. I believe that I have an appropriate level of expertise to confirm that it is of an acceptable scientific standard.

# Peer review: Referee response for version 1

**Published:** 2013-04-19

**Authors:** Jennifer N Slim

**Affiliations:** 1 Department of Medicine, San Antonio Military Medical Center, San Antonio, TX, USA

The Authors discuss hypertensive arteriosclerosis and hypothesize on extrinsic compressive forces from the expanding aneurysm as contributing to the acute ischemic changes noted in the brainstem. It would be interesting to learn more of the clinical course with improvement in blood pressure control and particularly if there were any significant reductions in aneurismal size on subsequent imaging.

I have read this submission. I believe that I have an appropriate level of expertise to confirm that it is of an acceptable scientific standard.
